# Prevalence of Vitamin A Deficiency in Pregnant and Lactating Women in the Republic of Congo

**DOI:** 10.3329/jhpn.v31i1.14746

**Published:** 2013-03

**Authors:** Claude Samba, Félicité Tchibindat, Bernard Gourmel, Patrick Houzé, Denis Malvy

**Affiliations:** ^1^Service des Maladies tropicales, Hospital Saint André, University Hospital Center of Bordeaux and Centre René Labusquière (Tropical Diseases Branch), EA 3677, University Victor Segalen Bordeaux 2, F-33076 Bordeaux, France;; ^2^CERMA (Centre d'Etudes et de Recherche Médecins d'Afrique), Brazzaville, Congo, ONG Médecins d'Afrique, Park 172, rue des fleurs, Quartier Ravin du Tchad, Brazzaville, Republique du Congo;; ^3^UNICEF Regional Office for West and Central Africa, BP 29720 Dakar, Senegal;; ^4^Laboratoire de Biochimie A, Hôpital Saint Louis,1 Avenue Claude Vellefaux, F-75011, Paris, France

**Keywords:** Dried blood spots, Lactating women, Pregnant women, Vitamin A deficiency, Congo

## Abstract

Vitamin A status in a sample of pregnant and lactating women living in several representative regions of Congo was assessed and compared between August and September 2004. This survey was conducted using a randomized two-stage cluster-sampling method with stratification on 90 clusters, each consisting of at least 15 women. Vitamin A status was determined in a total of 1,054 individuals, using the impression cytology with transfer (ICT) test, the modified relative dose response test (MRDR test) on dried blood spots (DBS), and clinical examination to detect signs of xerophthalmia. The clinical criterion defining vitamin A deficiency was the presence of active xerophthalmia (Bitot's spots [X1B]), active corneal disease), and/or night blindness (XN stage). The prevalence of clinical signs of stage XN and X1B xerophthalmia in the Republic of Congo was found to be 16% and 19% respectively. The prevalence of clinical signs (X1B) was greater in the rural north than in urban areas, with a gradient running from urban (5%) to rural area (33%); 27% of all the ICT tests showed that the subjects were suffering from vitamin A deficiency. The deficiency rates were significantly higher (p<0.001) in urban surroundings (Brazzaville) than in the rural northern regions. The biochemical MRDR test showed the presence of vitamin A deficiency (≥0.06) in 26% of the mothers in Brazzaville compared to 6% in the town of Kouilou; 44% of the women had retinol levels of <10 μg/dL in the rural north whereas these percentages were significantly lower in the urban areas surveyed (chi-square=62.30, p<0.001). A significant correlation was found to exist (p<0.001) between the ICT test and the MRDR test on DBS. In the population as a whole, 30% of the mothers suffering from malarial attack had abnormally low MRDR levels (≥0.06) compared to no malaria. The results of the present study confirm that vitamin A deficiency is a serious public-health issue in pregnant and lactating mothers in the Republic of Congo.

## INTRODUCTION

Vitamin A deficiency is a serious public-health issue in some countries with few resources, such as those in sub-Saharan Africa. Combating this problem in these countries has been a priority by the World Health Organization (1). We, therefore, used the innovative, efficient and reliable method called ‘modified relative dose response’ (MRDR) test on dried blood spots (DBS) for evaluating the retinol levels in pregnant and breastfeeding women exposed to the risk of vitamin A deficiency in the Republic of Congo (2,3,4).

## MATERIALS AND METHODS

### Study areas and setting

The study was carried out from August to September 2004 in Brazzaville and the town of Kouilou which are referred to as urban regions and in the Lekoumou, Likouala and plateau regions which are collectively referred to as the rural north region at the end of the dry season, a period when the vitamin A status of populations can be assumed to be at its lowest level. These regions were selected based on the accessibility of the villages, cooperation of village leaders, and the socioeconomic and ecological conditions. Three sets of areas were defined: the rural north area (30 clusters), Brazzaville (30 clusters), and Kouilou (30 clusters). One thousand fifty-four women in total were recruited in these areas: 365 subjects in the rural north, 348 in Brazzaville, and 341 in the town of Kouilou. The diet of these populations consists mainly of dark-green leafy vegetables, cassava tuber (the staple food), sweet potatoes, and bananas and has a relatively poor energy content. The protein supply is known to be extremely low in these regions. This surge is the sign of an early onset of the lean season—the toughest period for household members, particularly children, when nothing is left from the last harvest while the next harvest is due at early October (5,6).

**Figure:**
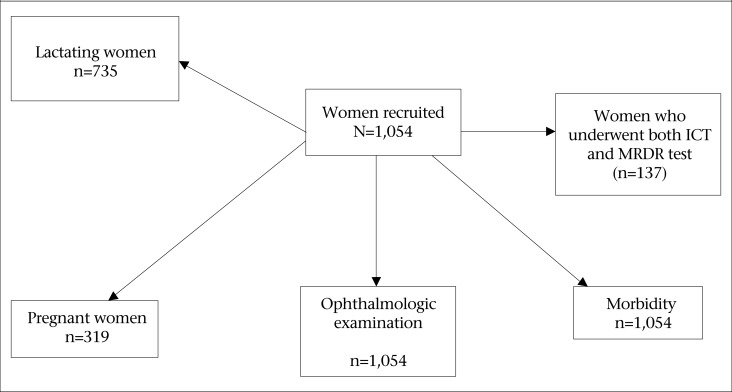
Flow-chart of enrolled pregnant and lactating women in the Republic of Congo

### Sampling methods

The population sample studied was recruited using a two-level, cross-sectional random cluster-sample design based on the procedures recommended by blindness prevention programme of the World Health Organization (1), with stratification based on agro-ecological factors. The information used for this purpose was based on the national nutrition and health survey (5,6) conducted in all eligible households irrespective of the nationality of their occupants and the length of time for which they had been living in the area. The figure shows a flow-chart of enrollment of these women. The sample of 1,054 pregnant and lactating women was assessed for the expected rate of vitamin A deficiency of 4%, an alpha error of 5%, and absolute accuracy requirements of 1%. The criterion defining vitamin A deficiency was the presence of active xerophthalmia (Bitot's spots, active corneal disease) and/or night blindness. At least fifteen women from each of the 90 villages (clusters) were randomly selected, accounting for a total of 1,054 individuals. If a cluster was very small for recruiting fifteen women, the nearest village was used for completing the cluster. No cluster effect was taken into consideration in determining the sample-size. Several subsamples were drawn randomly as described in the figure.

### Measurements and indicators

The data collected on each woman included socioeconomic status, morbidity, clinical ophthalmologic findings, histological findings, and the results of biochemical dried blood spot (DBS) tests (Table 1).

### Morbidity

Based on the results of the clinical and laboratory examinations, the women were divided into three groups: those having malarial attacks, fever-related current symptoms, and no malarial attacks. The malarial attack was confirmed for each woman by examination of a drop of blood or a simple blood smear. Women having fever-related current symptoms (headache, vomiting, and subjective sensations of fever) were identified by a family interview. The selection of the controls (apparently normal having no fever or malaria) was made in each household from the women who appeared to be in good health. Given the high incidence of malaria in the People's Republic of Congo, it is probable that these women may themselves be asymptomatic carriers of malaria.

**T1able 1.** Characteristics of the populations studied, based on the 2004 Congolese nutritional survey

Condition of women	N (%), 95% CI			
	Brazzaville (urban)	Kouilou (urban)	Rural north (northern forest)	All samples
Pregnant	110/348 (32.0), 27.0-37.0	62/341 (18.0), 13.8-22.2	147/365 (40.0), 34.9-45.1	319/1,054 (30.0), 27.2-32.8
Lactating	238/348 (68.0), 63.0-73.0	279/341 (82.0), 77.8-86.2	218/365 (60.0), 54.9-65.1	735/1,054 (70.0), 67.2-72.8
No occupational activity	268/348 (77.0), 72.5-81.5	248/341 (73.0), 68.2-78.0	286/365 (78.0), 74.0-82.3	802/1,054 (76.0), 73.4-79.0
Follow-up during pregnancy	310/348 (89.0), 85.7-92.3	289/341 (85.0), 81.1-88.9	295/365 (81.0), 76.9-85.1	894/1,054 (85.0), 82.8-87.2
Vitamin A capsules	112/348 (32.0), 27.0-37.0	74/341 (22.0), 17.5-26.5	120/365 (33.0), 28.1-37.9	306/1,054 (29.0), 26.2-31.8
Ocular infection	160/348 (46.0), 40.7-51.3	180/341 (53.0), 47.6-58.4	12/365 (3.0), 1.2-4.8	352/1,054 (33.0), 30.1-35.9
Night blindness (XN)	45/348 (13.0), 9.4-16.6	41/341 (12.0), 8.5-15.5	81/365 (22.0), 17.7-26.3	167/1,054 (16.0), 13.7-18.3
Xerophthalmia Bitot spots (X1B)	68/348 (19.0), 14.8-23.2	18/341 (5.0), 2.6-7.4	119/365 (33.0), 28.1-37.9	205/1,054 (19.0), 16.6-21.4
Malarial attacks	57/348 (16.0), 12.1-19.9	17/341 (5.0), 2.6-7.4	53/365 (15.0), 11.3-18.7	127/1,054 (12.0), 10.0-14.0
Fever-related symptoms	44/348 (13.0), 9.4-16.6	16/341 (5.0), 2.6-7.4	64/365 (18.0), 14.0-22.0	124/1,054 (12.0), 10.0-14.0

N (%)=Number (percentage) of the women, 95% CI=Confidence interval

### Ophthalmologic assessment

Ophthalmologic examination was performed on the entire sample (n=1,054) by trained ophthalmologists, using an X2.5 loupe with an electric torch. A history of night blindness (XN stage) was obtained by questioning mothers, using a vernacular term. The xerophthalmia outcome was diagnosed when Bitot's spots, specific active corneal lesions, or night blindness occurred. Vitamin A treatment (one capsule of 200,000 IU X 3) was given to all lactating mothers showing clinical signs of vitamin A deficiency, and tetracycline ointment was applied in case of ocular infection. Vitamin A treatment (one capsule of 10,000 IU daily for two weeks) was given to each pregnant woman showing suspected clinical signs of vitamin A deficiency (1). Any supplementary vitamin A capsules taken during the previous three months were also noted. Dispensaries were opened, and at least one nurse, one technician, and one fieldworker were present every day.

### Vitamin A status

#### Impression cytology with transfer

Impression cytology with transfer (ICT) test was performed on the entire sample. This test is based on the cellular differentiation induced by vitamin A. It makes use of cellulose acetate filter paper for sampling the cells of the conjunctiva of each eye. The technique of the test was modified by Luzeau and is currently called ‘impression cytology with transfer’ (7). The simplification involved a transfer of the conjunctival cells present on the sample paper to a glass-slide by light finger-pressure. We defined four stages on the basis of cytological criteria depending on the presence (sufficient status) or absence (deficient status) of goblet cells and the morphology of the epithelial cells (Table 2). In the case of deficiency and marginal minus stages, the impression cytology test was repeated one to three days later for confirmation. All of the slides were read on the spot. The slides were read again in double blind technique. All results from the ‘marginal minus’ and ‘deficient’ were examined again by optical microscopy.

**T1able 2.** Classification of the four cytological stages based on ICT tests

Presence or absence of goblet ells	Appearance of the epithelial cells	Stages*	Results
Present	Numerous, small, in clusters	N	Negative
Present	Numerous, more small than enlarged cells occurring separately	M+	Negative
Absent: mucin spots present	More enlarged, separate cells than small cells occurring in clusters	M-	Positive
Absent	Enlarged, separate	D	Positive

N=Normal; M+=Marginal plus;

M-=Marginal minus; D=Vitamin A-deficient;

*Based on impression cytology with transfer test

### Biochemical parameters

A subsample was drawn randomly in which one woman out of every 13 was subjected to a biochemical status assessment, using the modified relative dose response (MRDR) test and serum level of retinol (n=137, two in each cluster, except in the rural north). The MRDR on DBS in the rural north is not reported because these have not been done due to the lack of acetate 3,4-dehydroretinol (DRA) (vitamin A2). This reagent was unavailable in the field. Blood samples were collected from finger-pricks, following dried blood spots (DBS) technique as previously described (2). The MRDR test is an individual indicator of the liver retinal stores as previously described (8). The ratio between acetate 3,4-dehydroretinol (DRA) and retinol was calculated. A deficiency was defined as a ratio of ≥0.06.

### Statistical analysis

Data were recorded on standardized forms and reviewed for accuracy and completeness. Two teams worked in tandem in each region to collect information throughout a two-month period. Statistical comparisons were carried out using the chi-square test for categorical variables. Continuous data across groups were compared by performing analysis of variance and were normally distributed. Data were analyzed using the Epi Info software (version 6.04b).

### Ethics

The procedure used was approved by ethical committees of both French and Congolese Ministries of Health, and a consent form written in French and translated into the local language was signed by the mothers prior to the examination.

## RESULTS

### Population characteristics in various regions

One thousand fifty-four women participated in this survey. Pregnant women accounted for 30% (319/1,054) in total and lactating women for 70% (735/1,054) (Table 1).

### Socio-occupational characteristics

The socio-occupational characteristics of the subjects are presented in Table 2. In urban Brazzaville, 77.0% (268/348) of the surveyed women were unemployed at the time of the survey. In urban Kouilou, 73.0% (248/341) were unemployed at the time of the survey. In the rural north, 78.0% (286/365) were unemployed at the time of the survey; 89% (310/348) of the women living in urban Brazzaville were followed for medical check-up during their pregnancy at a hospital against 85% (289/341) and 81% (295/365) in urban Kouilou and the rural north respectively.

### Malarial morbidity

In urban Brazzaville, urban Kouilou, and the rural north, the pregnant and lactating women suffering from malarial attacks accounted for 16% (57/348), 5% (17/341), and 15% (53/365) respectively (Table 1). In the whole sample surveyed, about 12% (127/1,054) suffered from malarial attacks. In urban Brazzaville, 13% (44/348) of the pregnant and lactating women suffered from fever-related current symptoms against 5% (16/341) and 18% (64/365) in urban Kouilou and the rural north respectively. In all the samples combined, about 12% (124/1054) suffered from fever-related current symptoms.

Two hundred ninety-one patients in urban Brazzaville, 324 in urban Kouilou, and 312 in the rural north had no malarial attacks. In the whole sample surveyed, 927 women had no malarial attacks.

### Vitamin A supplementation coverage

The vitamin A supplementation coverage rate was 32% (112/348) in urban Brazzaville against 22% (74/341) and 33% (120/365) in urban Kouilou and the rural north respectively (Table 1). The vitamin A supplementation coverage rate was 29% (306/1,054) at the national level for women—a very low rate for a country where the needs are so great. This programme was interrupted by the civil war in these hardly-approachable regions.

### Vitamin A status

#### Clinical markers

The results of the clinical and ophthalmologic examinations are presented in Table 1. Interviews yielded the following data. The pregnant and lactating mothers who had a history of night blindness (XN) accounted for 13.0% (45/348), 12.0% (41/341), and 22.0% (81/365) in urban Brazzaville, urban Kouilou, and the rural north respectively. In Brazzaville, 19.0% (68/348) had Bitot's spots (X1B), followed by 5.0% (18/341) in Kouilou and 33.0% (119/365) in the rural north. No signs of stage X2 or X3 xerophthalmia were noted. The prevalence of clinical signs in the study population as a whole was as follows: Stage XN–16% (167/1,054) and Stage X1B–19% (205/1,054). Since 20 women had both, the overall prevalence of ocular infections in pregnant and lactating women was 33% (352/1,054).

### Histological markers

The results of the ICT test in terms of the four cytological classes defined in Table 2 are presented in Table 3; 27% (281/1,054) of the individuals were vitamin A-deficient; and 2.4% (25/1054) were rated marginal to poor, which indicated that they risked evolving to vitamin A deficiency status. The deficient ICT stage was significantly more frequent (p<0.001) among the pregnant and lactating women living in urban Brazzaville (33.0%, 117/348) than in urban Kouilou (23.0%, 78/341) and the rural north (23.6%, 86/365).

**T1able 3.** ICT and MRDR test results in pregnant and lactating Congolese women

Test	N (%), 95% CI			
	*Brazzaville (urban)	*Kouilou (urban)	*Rural north	All samples
*ICT				
Normal	222/348 (64.0), 59.0-69.0	253/341 (74.0), 69.3-78.5	255/365 (70.0), 45.0-49.0	730/1,054 (69.0), 66.0-72.0
Marginal+	7/348 (2.0), 0.5-3.5	1/341 (0.3), 0.0-0.9	10/365 (3.0), 4.0-6.0	18/1,054 (2.0), 0.9-2.5
Marginal-	2/348 (1.0), 0.6-1.4	9/341 (2.7), 2.2-3.2	14/365 (4.0), 6.0-8.0	25/1054 (2.4), 1.5-3.4
Deficient	117/348 (33.0), 29.0-39.0	78/341 (23.0), 18.5-27.5	86/365 (23.6), 19.1-28.1	281/1,054 (27.0), 24.3-29.7.0
All samples	348	341	365	1,054
**MRDR test				
≥0.06	10/39 (26.0), 12.0-40.0	3/55 (6.0), 5.0-6.0	nd	13/94 (14.0), 5.3-18.7
<0.06	29/39 (74.0), 60.0-88.0	52/55 (94.0), 93.0-94.0	nd	81/94 (86.0), 81.3-94.7
		N (%), Mean±SD		
**Retinol level (µg/dL)				
<10	5/39 (13.0), 7.1±1.9	14/55 (25.0), 8.9±1.9	19/43 (44.0), 6.45±2.35	38/137 (28.0), 7.5±2.05
10-20	11/39 (28.0), 13.1±2.1	24/55 (44.0), 16.07±2.1	10/43 (23.0), 14.55±2.65	45/137 (33.0), 14.5±2.3
>20	23/39 (59.0), 28.0±6.0	17/55 (31.0), 25.3±6.3	14/43 (37.0), 31.0±5.4	54/137 (39.0), 28.1±5.9

Chi-square=49, Degrees of freedom: ddl=6;

*p<0.001; Chi-square=62.30,

**p<0.001; CI=Confidence interval; ICT=Impression cytology with transfer test; nd=Not done; N (%)=Number (percentage) of the women

### Biochemical markers

Overall, 14.0% (13/94) of the results obtained from the women's MRDR tests on DBS were ≥0.06, reflecting the existence of serum retinol deficits in the hepatic reserves (Table 3). In urban Brazzaville, 26.0% (10/39) were deficient, which is significantly higher (p<0.001) than in urban Kouilou (6%) (3/55). The serum retinol deficits (<10 μg/dL) were present in 44.0% (19/43) of the individuals living in the rural north whereas this figure was 13.0% (5/39) in urban Brazzaville and 25.0% (14/55) in urban Kouilou. However, 33.0% (45/137) of the subjects were found to have serum retinol concentrations ranging from 10 to 20 μg/dL (14.5±2.3), reflecting the presence of subclinical vitamin A deficiency. A significant correlation was found to exist between the MRDR test results and the serum retinol concentrations (p<0.001).

### Correlation between ICT and MRDR tests in pregnant and lactating women

Vitamin A status given by the ICT test was deficient in 89.0% (34/38) of the mothers with MRDR test results of ≥0.06. A significant correlation was found to exist between the results of the MRDR test and those of the ICT test (chi-square=65.64, p<0.001) (Table 4). The sensitivity, specificity, and positive predictive values (PPV) of conjunctival impression cytology with transfer were calculated using MRDR on DBS as gold standards in Table 5.

### Correlation between MRDR tests and mala-rial attacks in pregnant and lactating women

The results of the MRDR tests were compared with the malarial morbidity rates.

Among the 29 pregnant and lactating mothers living in urban Brazzaville, who suffered from malarial attacks, 86.0 % (25/29) had abnormal MRDR levels (≥0.06) compared to 26.0% (10/39) of those with no malarial attacks (p<0.001); 83.0% (24/29) of malarial patients had serum retinol levels below 20 μg/dL compared to 41.0% (16/39) of those with no malarial attacks (chi-square=13.7, p=0.09). Among the 29 mothers who suffered from fever-related current symptoms, 63.0% (18/29) showed serum retinol levels below 20 μg/dL. In urban Kouilou, among the 63 mothers who suffered from malarial attacks, 95% (60/63) had abnormal MRDR test results, against 5.5% (3/55) of those with no malaria. Among the three of those who suffered from fever-related current symptoms, 5.0% (3/63) also had abnormal MRDR test results (≥0.06). At the national level, 30.0% (28/92) of the women patients who suffered from malarial attacks had abnormal MRDR test results compared to 14% (13/94) of those who had no malarial attacks. Among the 54 subjects who suffered from malarial attacks, 86.0% (54/63) living in urban Kouilou had serum retinol levels lower than 20 μg/dL compared to 69.0% (38/55) of the individuals who had no malarial attacks. Among the total 92 individuals who suffered from malarial attacks, 85.0% (78/92) had serum retinol concentrations below 20 μg/dL compared to 61.0% (83/137) of those with neither malaria nor fever; 83% (88/106) who suffered from fever-related current symptoms had abnormal MRDR test results (Table 6).

**T1able 4.** MRDR levels in pregnant Congolese women in the four ICT stages

Parameter	Urban Brazzaville		Urban Kouilou		Rural north		All samples	
ICT* stages	(N+ M+)	(M- + D)	(N+ M+)	(M- + D)	(N+ M+)	(M+ D)	(N+ M+)	(M- + D)
MRDR ≥0.06*	14	20	14	23	nd	nd	37	34
MRDR <0.06*	3	2	4	2	nd	nd	7	4
All samples	17	22	27	16	nd	nd	44	38
N° (%), 95% CI	14/17	20/22	23/27	14/16			37/44	34/38
	(82.0)	(91.0)	(85.0)	(87.5)	nd	nd	(84.0)	(89.0)
	61.0-100	78.0-100	71.3-9.0	71.0-100			73.0-95.0	79.0-99.0

nd=not done;

*Relationship between ICT stages and MRDR classes

**T1able 5.** Sensitivity, specificity, and positive predictive values of conjunctival impression cytology with transfer compared to MRDR in pregnant and lactating Congolese women

Conjunctival impression cytology	Urban Brazzaville		Urban Kouilou		All samples	
	MRDR	MRDR	MRDR	MRDR	MRDR	MRDR
	≥0.06	<0.06	≥0.06	<0.06	≥0.06	<0.06
Abnormal (M-,D)	20	2	14	2	34	4
Normal (M+,N)	14	3	23	4	37	7
Sensitivity	91% (20/22)		88% (14/16)		90% (34/38)	
	x100		x100		x100	
Specificity	15% (4/27)		15% (4/27)		16% (7/44)	
	x100		x100		x100	
Positive predictive value	59%		38%		90%	

D=Vitamin A-deficient;

M+=Marginal plus;

M-=Marginal minus; N=Normal

**T1able 6.** MRDR test on DBS versus malarial morbidity in Congolese women

Parameter	N (%), 95% CI			
	Urban Brazzaville	Urban Kouilou	Rural north	All samples
MRDR Test	*≥0.06	*≥0.06	*≥0.06	*≥0.06
*Malarial attacks	86% (25/29)	5.5% (3/55)	nd	30% (28/92)
	73.0-99.0	0.5-11.5		21.1-39.7
No malarial attacks	26% (10/39)	95% (60/63)	nd	14% (13/94)
	12.0-40.0	89.0-100		5.3-18.7
Fever-related current symptoms	62% (18/29)	5% (3/63)	nd	23% (21/92)
	44.0-80.0	0-10.4		14.5-31.5
**Retinol levels (µg/dL)	<20 µg/dL	<20 µg/dL	<20 µg/dL	<20 µg/dL
*Malarial attacks	83% (24/29)	86% (54/63)	0	85% (78/92)
	46.5-100.0	62.0-100.0		65.0-100.0
No Malarial attacks	41% (16/39)	69% (38/55)	67% (29/43)	61% (83/137)
	25.6-56.4	56.8-81.2	53.0-81.0	51.0-68.2
Fever-related current symptoms	63% (18/29)	97% (61/63)	64% (6/14)	83% (88/106)
	28.0-98.0	73.0-100.0	43.0-100.0	64.0-100.0

*Relationship between MRDR test and malarial attack;

**Relationship between retinol levels and malarial attack; nd=not done

## DISCUSSION

In 2011, the global food and nutrition situation was characterized by a critical level and inadequate food supplies. This reflects the current deterioration of the nutritional and vitamin A status in pregnant and lactating women of Congo since 2004. The current coverage of vitamin A supplementation remains low at the national level because of the ongoing civil war and the resulting socioeconomic instability, poverty, and inaccessibility to some areas. The current level of clinical xerophthalmia remains high since 2004 that required emergency response in 2012.

### Population

Since we included accessibility as a criterion for inclusion of a village, our data are certainly not representative of the entire country. However, it is by no means necessary for these data to be representative of the entire country for our survey to be quite valuable and that they are typical of the main ecologies present in the country.

### The dried blood spot (DBS) technique

The dried blood spot samples required for the MRDR tests were stored for three months before being analyzed. Appropriate precautions were taken during the storage of the samples, i.e. they were kept at a low temperature and protected from light. Under these conditions, DBS has been found to be stable for 18 months (9). In 2011, this technique has been used in India for assessment of serum retinol concentration in young children (10). In 2000, Craft Neal *et al*. (9) developed a method of DBS retinol determination, based on HPLC with spectrophotometric detection procedures, which is suitable for use on healthy adults. We have developed a high-performance liquid chromatography (HPLC) (2,3,4) with electrochemical detection for measuring DBS retinol. This technique can be useful and has been recommended. This tool can be applied in the context of countries with few resources, such as those in sub-Saharan Africa.

### MRDR on dried blood spots (DBS) method performed on pregnant and lactating women

In 2002, Tanumihardjo (8) reported that vitamin A deficiency is a problem in pregnant Indonesian women. The MRDR values were low in this population. These results are in line with those obtained in our study on the pregnant and lactating Congolese women. Thus, vitamin A deficiency is certainly a serious public-health problem in the Republic of Congo.

The method is linear up to 2.5 µM with a detection limit of 0.04 µM. Precision is below 10%, and DBS retinol recovery average is 90% (2). DBS is cheaper, and logistics are much simpler, less invasive as the authors describe, especially in rural surveys (2,3,4,9,10).

### Histological markers: ICT test performed on Congolese women

It is difficult to diagnose vitamin A status in the most severely-hit areas. Serum retinol is very expensive; the collection and care of samples in the field is very complex; the equipment needed (HPLC) are expensive and difficult to maintain and use correctly for serum retinol. Therefore, the impression cytology with transfer (ICT) test seems to be a useful complementary method of assessment for implementing mass population programmes in Central Africa because it is a simple-to-use and inexpensive means of determining the prevalence of vitamin A deficiency in this population.

This is the first study using the ICT test in Congolese women. This experience necessitates the comments that follow.

### Sensitivity, specificity, positive predictive value

The sensitivity and specificity of the ICT test were calculated when MRDR was used as the gold standard. We have compared the results of the ICT test with MRDR on DBS (≥0.06). These values show a risk of deficiency with low hepatic reserves. With respect to abnormal MRDR, the sensitivity, specificity, and the positive predictive value (PPV) are 90%, 16%, and 48% respectively in all women. We observed an increase in the sensitivity, which is the desired characteristic of a mass screening test with an MRDR threshold fixed at ≥0.06.

### MRDR test results in pregnant and lactating women suffering from malarial attacks

On average, 30.0% of the women suffering from malarial attacks included in this study were found to have abnormal MRDR levels; 83% (24/29) of the individuals living in urban Brazzaville and having malarial attacks had serum retinol concentrations below 20 μg/dL, reflecting subclinical vitamin A deficiency compared to those with no malaria (41%, 16/39). These data are consistent with those published from previous studies (11).

### MRDR test in comparison with the ICT test

The results of the MRDR tests indicated the presence of a deficiency (MRDR ≥0.06), which was defined as an abnormality by the ICT tests. The ICT results obtained in this study indicated the presence of subclinical vitamin A deficiency whereas the MRDR levels reflected greatly-depleted hepatic vitamin A reserves.

### Vitamin A supplementation coverage

Most rural and urban areas included in our study are socioeconomically deprived, and the youngest mothers had probably not been entirely covered by the vitamin A supplementation campaigns which were run before our survey took place. On the other hand, it is worth mentioning that the situation had improved in both rural north and the two urban areas since more than 29% of the inhabitants of the regions studied had been taking vitamin A capsules in the 3 months prior to our survey.

### Vitamin A status

#### Clinical markers

The socioeconomic and socio-occupational level of Congolese women was very low at the time of the study. The general level of hygiene and cleanliness left much to be desired. Most women included in this study had low household income and purchasing power. These unfavourable living conditions may be responsible for the high level of xerophthalmia (11,12,13). The values of the clinical markers of vitamin A deficiency obtained in our study were much higher than the threshold values established by WHO (1).

#### Night blindness

Night blindness (XN) is a subjective but reliable indicator of vitamin A deficiency (14,15,16). The prevalence of clinical signs of stage XN xerophthalmia showed a gradient running from the urban regions (Brazzaville and Kouilou—13.0% and 12.0% respectively) to the rural north (22.0%).

#### Bitot's spots

Bitot's spots are thought to be an early pathognomonic sign of vitamin A deficiency. The prevalence of stage X1B xerophthalmia, which has much longer-lasting effects, was higher in rural than in urban areas, with a decreasing gradient running from rural north to urban areas because of social underdevelopment.

### Conclusions

We suggest that the MRDR test on DBS is a good tool for conducting assessments of vitamin A status in sub-Saharan African settings. Based on our experience, it can be concluded that the impression cytology method with transfer is a good indicator of peripheral vitamin A deficiency in developing countries. It is less expensive and more convenient than the other available methods.

## ACKNOWLEDGEMENTS

This study was financed by UNICEF. We would like to thank Mrs. Régine Luzeau for her practical advice and Dr. Jessica Blanc for revising the English manuscript.
